# A Novel Phenomenological Constitutive Model for Semi-Crystalline Polymers Across a Wide Strain-Rate Range

**DOI:** 10.3390/polym17060762

**Published:** 2025-03-13

**Authors:** Yuxiang Zhang, Runqiang Chi, Shengjie Wang, Xuewen Zhang, Jiyue Si, Yuchen Zhao, Guangzhi Cui, Jun Ma

**Affiliations:** 1 Beijing Machine and Equipment Institute, Beijing 100854, China; naruto114@163.com (Y.Z.); youaresmart@126.com (S.W.); sijy@njust.edu.cn (J.S.); zyczky1120@163.com (Y.Z.); bokaw@163.com (G.C.); chinamajun@163.com (J.M.); 2 Hypervelocity Impact Research Center, Harbin Institute of Technology, Harbin 150080, China

**Keywords:** constitutive model, semi-crystalline polymer, strain rate, viscoelastic–viscoplastic

## Abstract

Focusing on the complex mechanical responses exhibited by semi-crystalline polymers under the coupled influences of strain hardening, strain-rate strengthening, and temperature softening, this paper proposes a phenomenological constitutive model employing a three-branch parallel structure. Using a hybrid global optimization algorithm, the optimal parameters for polypropylene were identified, attaining a coefficient of determination of 0.9834 and controlling the average absolute relative error within 6.4%. Moreover, the effectiveness of the proposed constitutive model was accurately validated through two material models from the LS-Dyna software 4.8.29 database, and the simulation results exhibited high consistency with the theoretical model. This study provides a high-confidence material model suitable for high-strain-rate simulation scenarios.

## 1. Introduction

Polymer materials, owing to their superior characteristics, including light mass, outstanding ductility, ease of processing, and low cost, have been widely applied in popular fields such as new energy batteries, precision sensors, the automotive industry, and aerospace [1,2,3,4,5]. Particularly in the field of space debris protection, Whipple shields made from polymer materials exhibit superior protective performance compared to traditional aluminum alloys [6,7,8]. Given the high costs and limitations of experimental methods for hypervelocity impact testing, the performance of new protective structures and materials is often validated through numerical simulation methods. To guarantee the accuracy of numerical simulation outcomes, it is crucial to establish an accurate and applicable material constitutive model [9]. This model must not only precisely characterize the nonlinear mechanical responses of materials, but also meet the requirements for computational efficiency optimization and software compatibility, enabling seamless integration with existing simulation algorithms and avoiding the risks of computational failure and numerical divergence.

Obtaining fundamental mechanical data of materials is a necessary prerequisite for establishing constitutive models and determining the parameters of these models; however, due to the limitations of experimental equipment, it is extremely difficult to obtain material data under hypervelocity impact conditions (with strain rates exceeding 10^6^ s⁻¹). As a result, existing data are often used for theoretical extrapolation. The split-Hopkinson pressure bar (SHPB) method is currently among the most widely utilized experimental techniques for characterizing material behavior under high-strain-rate conditions [10]. Okereke et al. [11,12,13,14] employed this technique to obtain the uniaxial compressive stress-strain curves of various polymer materials at strain rates around 10^4^ s^−1^. Similarly, Xu et al. [15,16] utilized a comparable approach to determine the corresponding tensile stress and strain relationships. Furthermore, Vuoristo [17,18,19] incorporated temperature control devices into their experimental setups to obtain the mechanical response curves of polymers under different ambient temperature conditions. By leveraging the time–temperature superposition principle [20,21], the mechanical behavior of polymers can be extrapolated to a broader strain-rate range based on the obtained results; however, the accuracy and reliability of this extrapolation method remain subjects of ongoing debate and require further validation. In addition, Zhang et al. [22,23,24] employed a light gas gun to investigate the transient deformation responses of materials such as polypropylene and polycarbonate at even higher strain rates. Despite its advantages in achieving high strain rates, this method is inherently limited in its ability to capture detailed material behavior during the deformation process.

There is a substantial amount of research on constitutive models for polymer materials; however, models for strain rates exceeding 10^4^ s^−1^ still require improvements in terms of accuracy and general applicability. Additionally, the existing models are relatively complex and difficult to directly apply in simulation software. Holmes et al. [25,26,27] advanced the field by proposing various forms of viscoelastic–viscoplastic models, which were fundamentally based on the Maxwell model [28] and the Voigt model [29]. While these models are capable of accurately capturing the mechanical responses under varying strain-rate levels, they still exhibit certain limitations in terms of predictive accuracy. Pouriayevali et al. [30,31,32] extended the scope of this research by incorporating the influence of environmental temperature into the constitutive framework; through the refinement of the functional relationship between model parameters and temperature, they significantly broadened the applicability of these models, enabling more accurate predictions of material behavior under varying thermal conditions. Moreover, Okereke et al. [33,34] adopted a physically based approach to constitutive modeling, integrating the molecular physical properties of polymeric materials into their formulations. Its validity was confirmed through comparison with experimental results, demonstrating its ability to accurately describe the materials’ mechanical behavior. To further validate the practical applicability of these constitutive models, Xu et al. [35,36] conducted a study employing numerical simulation techniques to investigate hypervelocity impact processes, which provided empirical evidence of the model’s effectiveness.

Existing constitutive models for polymer materials have insufficient accuracy under high-strain-rate conditions and are relatively complex, making them difficult to directly apply in numerical simulation software. This paper proposes a three-chain parallel constitutive model for semi-crystalline polymer materials over a broad strain-rate spectrum, and determines the optimal model parameters for polypropylene using a hybrid global optimization algorithm. The theoretical results obtained are in close alignment with experimental data. The model structure is able to be directly integrated into the algorithm framework of numerical simulation software, providing a high-confidence material model for subsequent hypervelocity impact simulations of polymer materials and offering an effective technical means with which to improve the performance of spacecraft protective structures.

## 2. Materials and Methods

### 2.1. Materials

**Figure 1:**
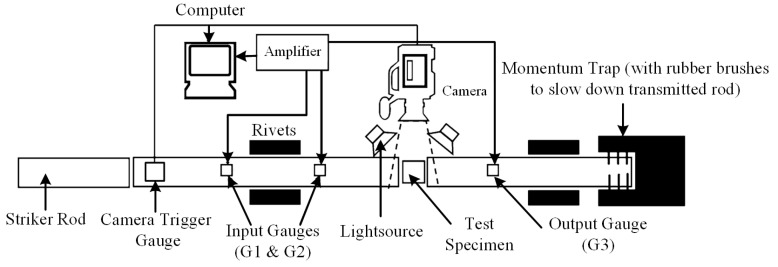
Schematic diagram of the SHPB.

Seeing the tests in Ref. [11], dynamic compression experiments on three types of polypropylene materials were conducted by using an SHPB testing apparatus. The SHPB device mainly consists of a striker rod, an incident rod, a transmitted rod, and testing devices. The schematic diagram of the device is shown in Figure 1, and the specific experimental details can be found in Refs. [37,38]. The materials of the striker rod, incident rod, and transmitted rod in the device are all martensitic steel, with dimensions of Ø16 mm × 250 mm, Ø15 mm × 500 mm, and Ø15 mm × 459 mm, respectively. Strain gauges G1, G2, and G3 were attached at 308 mm, 40 mm from the sample on the incident rod, and 40 mm from the sample on the transmitted rod. The compression test samples are two types of cylinders with a length-to-diameter ratio of 0.3, the first type with dimensions of Ø5 mm × 1.5 mm, and the second type with dimensions of Ø10 mm × 3 mm.

**Figure 2:**
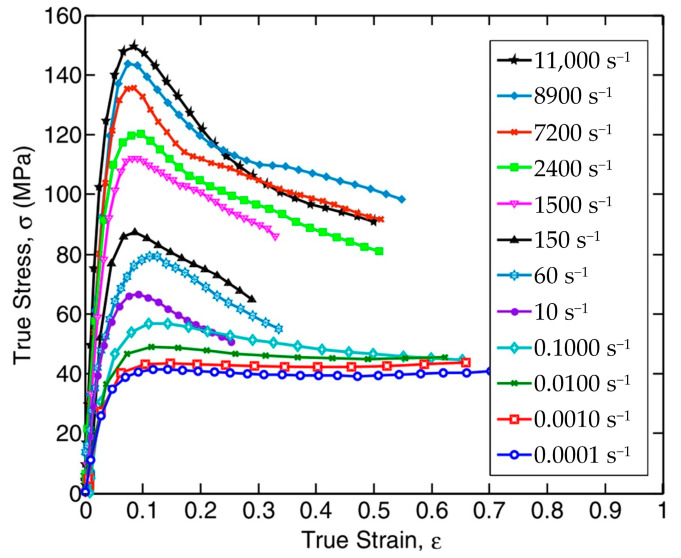
The compressive response of the polypropylene.

In this paper, the experimental results of the ICI sample were utilized for model establishment and parameter fitting. Figure 2 displays the stress and strain relationships of the polypropylene within the strain-rate spectrum of 0.0001 s^−1^ to 11,000 s^−1^. The polypropylene was previously manufactured by the ICI (Imperial Chemical Industries, London, UK) under the propathene grade GWM22. Its density is (0.9078 ± 0.001) g/cm^3^, and the crystallinity is (65 ± 1) %.

In Figure 2, the stress–strain curves corresponding to strain rates below 1 s^−1^ are related to quasi-static compression tests, while those above 1 s^−1^ are associated with dynamic compression tests. The deformation behavior of polypropylene during compression can be classified into two distinct phases. In the early deformation step, the material exhibits viscoelastic characteristics. And as strain increases, the slope of the stress-strain curve progressively diminishes. At this stage, the deformation of the material is mainly caused by the local vibration and slight displacement of the molecular chains. The work performed by the external force is almost entirely stored as reversible elastic potential energy within the material. Since there is little overall conformational change in the molecular chains and minimal internal friction or slip, energy dissipation is very low and insufficient to cause a temperature rise in the material [39].

Once a certain threshold strain is exceeded, a noticeable inflection point appears on the curve, indicating the transition of the material into the viscoplastic stage. After entering the viscoplastic stage, irreversible slip, disentanglement, or fracture occurs between the molecular chains, leading to increased internal friction and the production of a significant amount of heat. Meanwhile, plastic deformation is accompanied by the proliferation and movement of dislocations, and the resistance generated by dislocation interactions hinders energy dissipation, making it difficult for the heat generated by plastic deformation to dissipate, resulting in a noticeable temperature rise [40]. The temperature increase enhances the movement of the molecular chains, decreases the material’s viscosity, and thus lowers the resistance to chain segment sliding and disentanglement. This means that the material’s ability to resist deformation decreases [41]. When the trend in stress reduction caused by the temperature rise surpasses the strain-hardening result, the material’s stress will decrease with increasing strain, as observed macroscopically.

### 2.2. Model Formulation

#### 2.2.1. Viscoelastic–Viscoplastic Model

**Figure 3:**
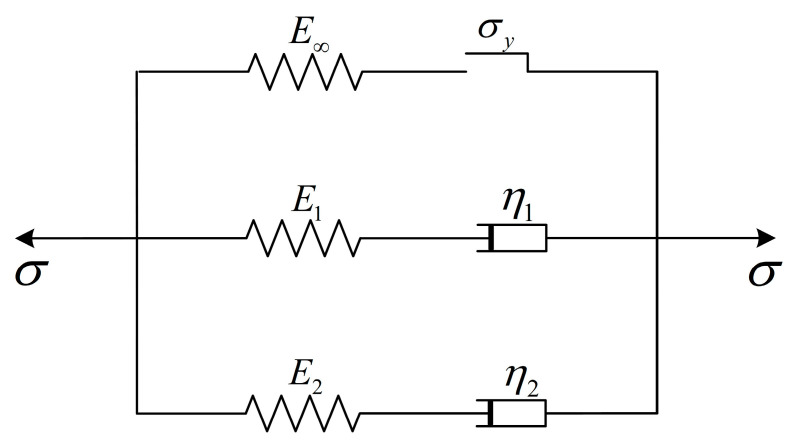
Schematic diagram of the viscoelastic–viscoplastic model.

The model is based on the generalized Maxwell model, with an additional non-Maxwell branch in parallel, as shown in Figure 3. The non-Maxwell branch consists of an elastic element that is strain-rate-independent but temperature-dependent, and a frictional slider in series. The generalized Maxwell branch consists of two parallel standard Maxwell branches. The development of the model includes both the polymer’s physical structure and the software compatibility.

In terms of physical structure, semi-crystalline polymers consist of crystalline and amorphous regions. The crystalline region typically exhibits high tensile strength and a high modulus, but due to its highly ordered and rigid crystalline structure, it has poor resistance to deformation and shows brittle characteristics. At the same time, the crystalline region has higher thermal resistance, with relatively small temperature-softening effects. In contrast, the amorphous region has low strength, high toughness, and thermal sensitivity [42,43]. The model uses different branches to represent the different physical structures within the polymers. The non-Maxwell branch represents the mechanical responses of the entangled molecular network in the semi-crystalline polymer, while the two Maxwell branches correspond to the mechanical behavior of the crystalline/rigid amorphous phase and the amorphous phase in the polymer.

In terms of software compatibility, many viscoelastic–viscoplastic constitutive equations in commonly used simulation software (e.g., LS-Dyna 4.8.29) are based on the generalized Maxwell model framework, and the proposed model form aligns with the intrinsic computational rules of the software. Alternatively, the count of branches in the model is relatively small, only three, which ensures the accuracy of the model while significantly reducing the computational load when embedded into the software, thus improving the stability of the simulation process.

#### 2.2.2. Viscoelastic Part

When the material is in the viscoelastic stage, the friction slider in the model can be neglected. The total deviatoric stress experienced by the material, $\sigma^{ve}$, can be represented as the total of the deviatoric stresses of the three branches. The total elastic strain, $\varepsilon^{ve}$, is equal to the strain in each branch, specifically as follows:

(1) $$\sigma^{ve}=\sigma_{e}^{ve}+\sum_{i=1}^{2}\sigma_{M_{i}}^{ve}$$

(2) $$\varepsilon^{ve}=\varepsilon_{e}^{ve}=\varepsilon_{M_{i}}^{ve}$$

where $\sigma_{e}^{ve}$, $\varepsilon_{e}^{ve}$, $\sigma_{M_{i}}^{ve}$, and $\varepsilon_{M_{i}}^{ve}$ represent the deviatoric stress and strain of the two types of branches, respectively.

For the non-Maxwell branch, stress can be expressed as follows:

(3) $$\sigma_{e}^{ve}=E_{\infty }^{ve}\varepsilon^{ve}$$

For the Maxwell branch, stress can be expressed as follows:

(4) $$\sigma_{M_{i}}^{ve}=E_{i}^{ve}(\varepsilon_{M_{i}}^{ve}-\varepsilon_{v_{i}}^{ve})$$

(5) $${\dot{\varepsilon }}_{v_{i}}^{ve}=\frac{1}{\eta_{i}^{ve}}\sigma_{M_{i}}^{ve}$$

where $E_{\infty }^{ve}$ and $E_{i}^{ve}$ are the elastic moduli of the elastic elements in the non-Maxwell and Maxwell branches, respectively. $\varepsilon_{v_{i}}^{ve}$, ${\dot{\varepsilon }}_{v_{i}}^{ve}$ and $\eta_{i}^{ve}$ are the strain, the strain rate, and the viscosity of the viscous elements, respectively.

When the material is subjected to pressure at a constant strain rate, $\dot{\varepsilon }=const$, taking the derivative of Equation (4) and substituting Equation (5) yields the following:

(6) $${\dot{\sigma }}_{M_{i}}^{ve}=E_{i}^{ve}(\dot{\varepsilon }-\frac{\sigma_{M_{i}}^{ve}}{\eta_{i}^{ve}})$$

Let us multiply both sides of Equation (6) by the integrating factor $e^{t/\theta_{i}^{ve}}$, and rearrange the equation into the form of an exact differential equation:

(7) $$\frac{d}{dt}(\sigma_{M_{i}}^{ve}e^{t/\theta_{i}^{ve}})=E_{i}^{ve}\dot{\varepsilon }e^{t/\theta_{i}^{ve}}$$

where t is the deformation time and $\theta_{i}^{ve}=\eta_{i}^{ve}/E_{i}^{ve}$ is the relaxation time of the branch. The relaxation time is a characteristic time that measures the transition of the material from elastic to viscous behavior, and reflects the response speed of the material’s internal structure to instantaneous stress [44].

Furthermore, we solve Equation (7) using the method of integration, and by substituting the initial value, $t=0,\;\sigma_{M_{i}}^{ve}=0$, the Maxwell branch deviatoric stress is obtained as follows:

(8) $$\sigma_{M_{i}}^{ve}=\eta_{i}^{ve}\dot{\varepsilon }[1-\exp(-\frac{t}{\theta_{i}^{ve}})]$$

As depicted in Figure 2, polypropylene exhibits a pronounced strain-rate-hardening effect during the viscoelastic stage. Several models have been proposed to characterize this effect, among which the Cowper-Symonds model [45] is widely used due to its simple form, high accuracy, and broad applicability in simulation software. Therefore, in this study, the model is constructed upon the C–S model, where viscosity and relaxation time are expressed as power–law dependencies on the strain rate, specifically as follows:

(9) $$\eta_{i}^{ve}(\dot{\varepsilon })=\eta_{i_{0}}(\frac{\dot{\varepsilon }}{{\dot{\varepsilon }}_{0}}{)}^{-\alpha_{i}}$$

(10) $$\theta_{i}^{ve}(\dot{\varepsilon })=\theta_{i_{0}}(\frac{\dot{\varepsilon }}{{\dot{\varepsilon }}_{0}}{)}^{-\beta_{i}}$$

where $\eta_{i_{0}}$, $\theta_{i_{0}}$, and ${\dot{\varepsilon }}_{0}$ are the reference viscosity, reference relaxation time, and reference strain rate, respectively; $\alpha_{i}$ and $\beta_{i}$ are the strain-rate coefficients.

Finally, by substituting the results from Equations (9) and (10) into Equation (8), the deviatoric stress of the Maxwell branch under different strain-rate conditions during the viscoelastic stage is derived as follows:

(11) $$\sigma_{M_{i}}^{ve}=\eta_{i_{0}}(\frac{\dot{\varepsilon }}{{\dot{\varepsilon }}_{0}}{)}^{-\alpha_{i}}\dot{\varepsilon }[1-\exp(-\frac{t}{\theta_{i_{0}}(\frac{\dot{\varepsilon }}{{\dot{\varepsilon }}_{0}}{)}^{-\beta_{i}}})]$$

In the viscoelastic model, strain, and strain rate are taken as independent variables, while stress is the dependent variable. The model includes nine unknown parameters: elastic modulus, $E_{\infty }^{ve}$; reference viscosity, $\eta_{i_{0}}$; reference relaxation time, $\theta_{i_{0}}$; and strain-rate coefficient, $\alpha_{i}$, $\beta_{i}(i=1,2)$. These parameters remain constant in the model and are unaffected by changes in strain and strain rate. Therefore, if at least nine sets of strain, strain rate, and corresponding stress values are known, these parameters can be determined by solving the explicit system of equations. Furthermore, the larger the amount of data used for fitting, the more accurate the obtained parameters will be.

#### 2.2.3. Viscoplastic Part

To accurately describe the mechanical behavior of polymers in the viscoplastic stage, it is essential to establish precise yield stress, $\sigma_{y}$, evolution equations. As the threshold that separates the material’s viscoelastic and viscoplastic stages, the accuracy of the yield stress directly determines the predictive capability of the entire constitutive model for the viscoplastic deformation mechanism and overall mechanical behavior. This paper adopts the Ree–Eyring theoretical model [46] to describe this critical parameter. The advantage of this model lies in its explanation of the correlation between yield stress and strain rate from a molecular dynamics perspective. By establishing a link between the thermally activated motion of polymer chain segments and macroscopic mechanical responses, the rate-dependent yield characteristics driven by the unique microscopic mechanisms of polymer materials and complex fluids are revealed. The mathematical formulation of the model is presented as follows:

(12) $$\sigma_{y}=\sigma_{0}+\frac{2kT}{V}\sinh^{-1}{\left(\frac{\dot{\varepsilon }}{{\dot{\varepsilon }}_{0}}\right)}^{1/\gamma }$$

where $\sigma_{0}$ represents the reference yield stress, k is the Boltzmann constant, T denotes the material temperature, V is the activation volume, and γ is a fitting coefficient.

After the material enters the viscoplastic deformation stage, a significant portion of the plastic work is converted into internal energy, leading to an increase in the material’s temperature. Compared to metallic materials, semi-crystalline polymers have a lower degree of molecular arrangement order and relatively weaker intermolecular forces. The increase in temperature greatly intensifies the relative motion of polymer chains, resulting in a significant decrease in mechanical properties [42]. As shown in Figure 2, after polypropylene enters the viscoplastic stage, the material stress gradually decreases with the increase in strain, and its temperature-softening effect is significantly stronger than the strain-hardening and the strain-rate-hardening effects.

The Johnson–Cook model [47] is particularly suitable for calculating the mechanical response of materials under high-strain-rate conditions, such as hypervelocity impact and explosion. At the same time, the model and its various modified forms have been incorporated into commonly used simulation software. Therefore, in this paper, the temperature term in the J–C model is modified, and the viscosity as well as the relaxation time in the Maxwell branch are defined as temperature functions in this form. Additionally, the elastic modulus in the non-Maxwell branch is defined as a linear function of temperature, as detailed below:

(13) $$E_{\infty }^{vp}(T)=E_{\infty_{0}}+a\times (T-T_{0})$$

(14) $$\eta_{i}^{vp}(\dot{\varepsilon },T)=\eta_{i_{0}}(\frac{\dot{\varepsilon }}{{\dot{\varepsilon }}_{0}}{)}^{-\alpha_{i}}\times b_{i}{\left(\frac{T-T_{0}}{T_{m}-T_{0}}\right)}^{c_{i}}$$

(15) $$\theta_{i}^{vp}(\dot{\varepsilon },T)=\theta_{i_{0}}(\frac{\dot{\varepsilon }}{{\dot{\varepsilon }}_{0}}{)}^{-\beta_{i}}\times d_{i}{\left(\frac{T-T_{0}}{T_{m}-T_{0}}\right)}^{e_{i}}$$

where $T_{0}$ and $T_{m}$ represent the initial temperature and melting temperature of the material, respectively. $E_{\infty_{0}}$ is the reference elastic modulus, while a, $b_{i}$, $c_{i}$, $d_{i}$, and $e_{i}$ are temperature coefficients.

In accordance with the first law of thermodynamics, the temperature increase caused by plastic deformation of the material is as follows [48]:

(16) $$T=T_{0}+\frac{\beta_{p}}{\rho \times Cv}\int \sigma^{vp}d\varepsilon^{vp}$$

where $\beta_{p}$ is the Taylor–Quinney factor [49], ρ and Cv represent the material density and specific heat capacity at a constant volume, respectively.

Compared to the viscoelastic model, the viscoplastic model adds temperature coefficients, a, $b_{i}$, $c_{i}$, $d_{i}$, and $e_{i}$, and material temperature, T, to the existing parameters. While the temperature coefficients remain constant in the equation, the material temperature changes with strain and is not constant in the equation. Therefore, during the solution process, the original equation needs to be converted into a differential equation form. By discretizing the strain values, the material temperature at different intervals can be obtained. The material temperature is then iteratively updated during the solution process, gradually determining the temperature coefficients. In this paper, the implicit Euler method [50] is specifically used. It is assumed that during the time interval, $\left[t_{n},t_{n+1}\right]$, the viscoplastic strain and temperature changes in the material are εvpΔ(tn+1) and TΔ(tn+1), respectively. The changes in the deviatoric stress for the non-Maxwell, σevpΔ(tn+1), and Maxwell, σMivpΔ(tn+1), branches can be expressed as follows:

(17) σevpΔ(tn+1)=(E∞0+a×TΔ(tn+1))×εvpΔ(tn+1)

(18) σMivpΔ(tn+1)=ηi0(ε˙ε˙0)−αi×bi(TΔ(tn+1)Tm−T0)ciε˙[1−exp(−tΔθi0(ε˙ε˙0)−βi×di(TΔ(tn+1)Tm−T0)ei)]

The deviatoric stress of the material $\sigma^{vp}(t_{n+1})$ can be expressed as follows:

(19) σevp(tn+1)=σevp(tn)+σevpΔ(tn+1)

(20) σMivp(tn+1)=σMivp(tn)+σMivpΔ(tn+1)

(21) $$\sigma^{vp}(t_{n+1})=\sigma_{e}^{vp}(t_{n+1})+\sum_{i=1}^{2}\sigma_{M_{i}}^{vp}(t_{n+1})$$

### 2.3. Numerical Simulation Model

To validate the feasibility of the proposed model within the simulation software, this study employed LS-Dyna 4.8.29 to perform a numerical simulation of the SHPB experiment on polypropylene materials. Compared with embedding the constitutive model directly into the software via secondary development, this paper instead employs the built-in material models MAT_89 and MAT_224 from the software library for the simulation. Using these existing material models can significantly enhance work efficiency and reduce the time required for programming and debugging, while also helping to ensure software stability and lower the risk of errors.

Regarding model selection, MAT_89 uses table definitions to represent the material’s full strain response under various strain rates, capturing both nonlinear elastic and nonlinear plastic behavior. This makes it particularly suitable for scenarios involving smaller deformations, high nonlinearity, and the need to track the entire deformation process. Although MAT_224 adopts a linear representation in the elastic phase, it accounts for the coupling of temperature and strain rate in the plastic phase and can be used in conjunction with an equation of state and multiple failure criteria. As a result, it is well suited to situations with high strain, thermo-mechanical coupling, and a primary focus on material plastic deformation and failure, such as hypervelocity impacts.

**Figure 4:**
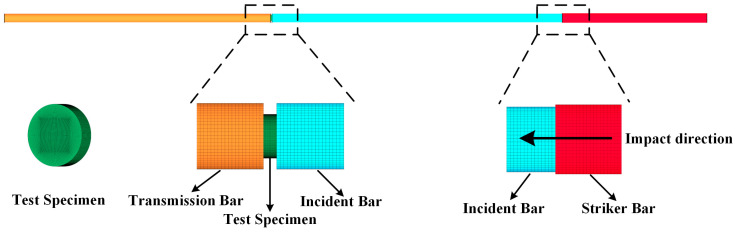
SHPB finite element model.

As shown in Figure 4, the simulation model includes the striker bar, incident bar, transmission bar, and test specimen, with dimensions matching those of the actual experiment. The bars are modeled using an elastic material model, characterized by an elastic modulus of 210 GPa, a Poisson’s ratio of 0.3, and a density of 7.8 g/cm^3^. The simulation mesh consists of hexahedral elements, with a mesh size of 0.8 mm for the bars and 0.1 mm for the test specimen. To address the issues of element penetration and distortion caused by the modulus difference between the bars and the sample, both contact and hourglass controls were defined. The contact between the sample and the bars was set up using the keyword “CONTACT_AUTOMATIC_SURFACE_TO_SURFACE” with the parameter “SOFT = 2”. Under “CONTROL_CONTACT”, the contact stiffness was set by specifying “SLSFAC = 1”. For the keyword “HOURGLASS”, the type was defined as “IHQ = 4, QM = 0.1”.

## 3. Results

The hybrid GlobalSearch optimization algorithm [51] generates a large number of initial points randomly and combines them with local optimization algorithms for multi-point iterative searching. Finally, the global optimal solution is obtained by comparing the search results of all the points. This algorithm addresses the problem of becoming trapped in local optimal solutions while ensuring computational efficiency, making it especially suitable for solving parameter identification problems with complex nonlinear dynamic behaviors and strongly non-convex objective functions. In this study, this method is employed to determine the optimal parameters of the yield stress, viscoelastic, and viscoplastic parts in the constitutive model.

Before fitting the yield stress parameters, it is necessary to define the selection of the yield stress point. For semi-crystalline polymers without a clear inflection point in the stress–strain curve during the deformation stage, there is no unified definition of the yield point. Common methods for determining the yield stress of materials under these conditions include the definition method, plotting method, equal energy method, and residual plastic deformation method [52]. In this study, following the definition method, the maximum stress observed throughout the entire deformation stage is identified as the yield point. The stress and strain values at this point are then utilized as the yield stress and yield strain for parameter fitting at the specified strain rate. The detailed fitting results are presented in Table 1.

**Figure 5:**
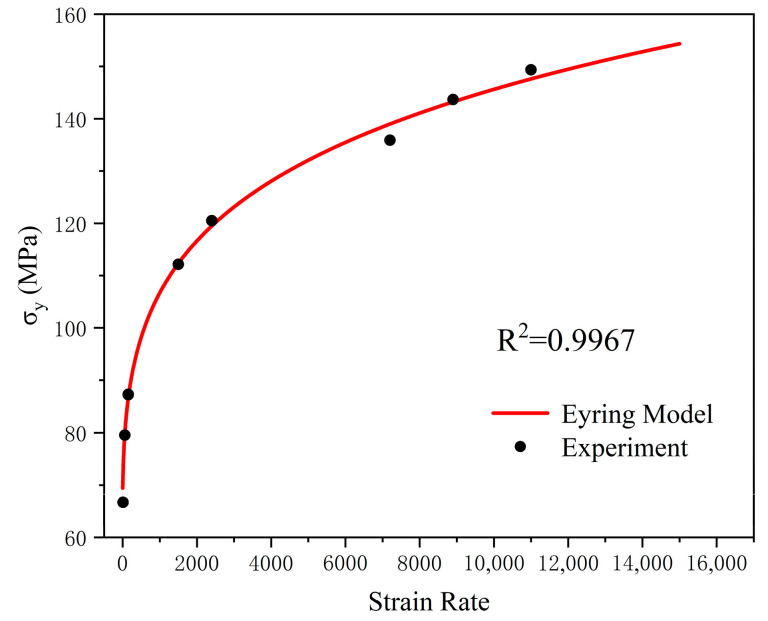
Comparison between the theoretical yield stress calculated based on the Ree–Eyring model and the experimental results.

Figure 5 presents a comparison between the theoretical yield stress calculated based on the Eyring model and the experimental results. The correlation coefficient of the model is 0.9967, which demonstrates that the theoretical calculations can accurately depict the effect of strain rate on the material’s yield stress.

In the constitutive model proposed in this paper, the entire deformation process of the material is divided at the yield point into two parts: viscoelastic and viscoplastic. When solving for the model parameters, one must first determine the viscoelastic parameters, which then serve as the basis for determining the viscoplastic parameters. Finally, these two parts are joined at the yield point; therefore, the predicted result at the conclusion of the viscoelastic segment (i.e., at the yield point) directly serves as the initial condition for the viscoplastic segment, and its accuracy decisively affects the predictive accuracy of both the viscoplastic segment and the entire model.

**Figure 6:**
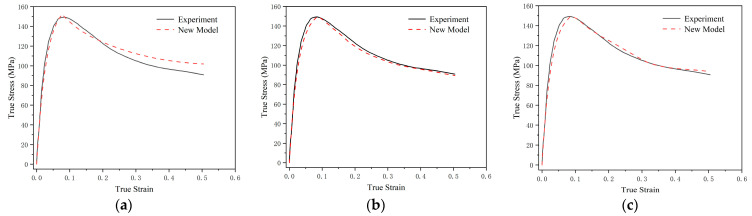
Comparison of predicted results under different fitting weights at a strain rate of 11,000 s^−1^. (**a**) 0; (**b**) 20; and (**c**) 100.

To improve prediction accuracy at this critical point, the fitting weight for that point can be increased during data fitting, prompting the algorithm to prioritize its accuracy. Table 2 presents the coefficients of determination for each deformation stage under different weight settings. The results show that, without adjusting the weight, the model provides relatively high predictive accuracy for both the viscoelastic segment and the overall deformation, but has comparatively lower accuracy for the viscoplastic segment. As the weight value increases, the accuracy of the viscoplastic segment improves, whereas the accuracy of the viscoelastic segment and overall deformation decreases. As can be seen more intuitively from Figure 6, there is a marked difference in prediction accuracy across the various deformation stages under different weight values.

Adjusting the fitting weight at the yield point directly influences the prediction accuracy of the model for different deformation stages. Since the proposed model is primarily intended for numerical simulations involving high strain rates and large deformations, such as hypervelocity impacts, it is necessary to ensure overall model accuracy while placing priority on the viscoplastic segment and high-strain-rate conditions. Taking various factors into account, a weight of 20 was ultimately selected for the fitting results. The parameters obtained from the fitting of the viscoelastic segment are shown in Table 3, and those obtained from the fitting of the viscoplastic segment are shown in Table 4.

**Figure 7:**
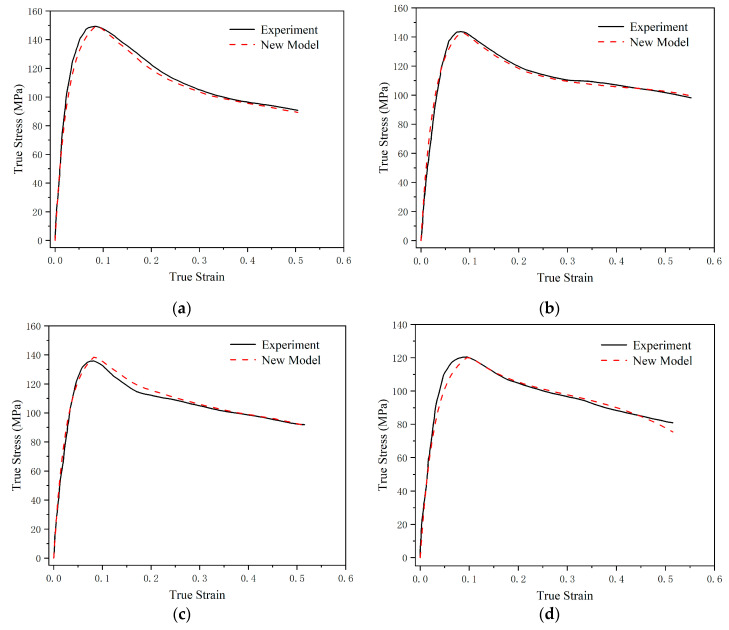
Comparison curves of the experimental data and theoretical prediction results for the material’s dynamic mechanical response within the strain-rate range of 2400 s^−1^ to 11,000 s^−1^. (**a**) 11,000 s^−1^; (**b**) 8900 s^−1^; (**c**) 7200 s^−1^; and (**d**) 2400 s^−1^.

Figure 7 presents the comparison curves of the experimental data and theoretical prediction results for the material’s dynamic mechanical responses within the strain-rate range of 2400 s^−1^ to 11,000 s^−1^. Within the high-strain-rate region, the predictive curves of the constitutive model show strong consistency with the experimental data. The theoretical results not only accurately describe the trend in the material’s mechanical responses but also have very small errors in the specific values compared with the experimental results, with only relatively larger errors in the viscoelastic part at the end of the strain rate of 2400 s^−1^.

**Figure 8:**
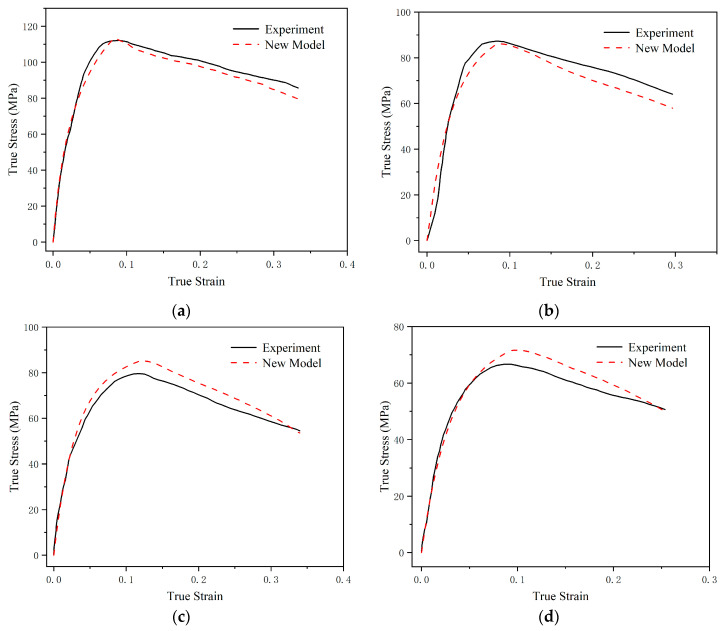
Comparison curves of the experimental data and theoretical prediction results for the material’s dynamic mechanical response within the strain-rate range of 10 s^−1^ to 1500 s^−1^. (**a**) 1500 s^−1^; (**b**) 150 s^−1^; (**c**) 60 s^−1^; and (**d**) 10 s^−1^.

Figure 8 presents the comparative curves of the experimental data and theoretical predictions for the material’s dynamic mechanical response within the strain-rate range of 10 s^−1^ to 1500 s^−1^. In the low-strain-rate region, the overall trend in the theoretical results is consistent with the experimental data, but the accuracy is relatively lower compared to the high-strain-rate region. There are three main reasons for the errors: First, experimental equipment and methods may introduce certain errors during data collection. For example, in the viscoelastic segment experimental data at a strain rate of 150 s^−1^, the curve slope shows a change from increasing to decreasing to increasing again, which clearly does not align with the material’s physical properties. Second, the proposed constitutive equation and parameter fitting method inevitably have certain limitations, making it difficult to fully replicate the experimental data. Lastly, as mentioned earlier, deviations in the data at the yield point can affect the accuracy of the entire plastic segment. For instance, at strain rates of 60 s^−1^ and 10 s^−1^, the higher-than-expected yield point results lead to the prediction of the entire plastic segment being higher than the experimental results.

## 4. Discussion

### 4.1. Evaluation of the Model

To further analyze the accuracy and reliability of the constitutive model proposed in this paper, the absolute relative error (ARE), the average absolute relative error (AARE), and the correlation coefficient (R^2^) of the predicted values were calculated, respectively:

(22) $$\mathrm{ARE}(\%)=\left|\frac{\sigma_{\exp}-\sigma_{Model}}{\sigma_{\exp}}\right|\times 100\%$$

(23) $$\mathrm{AARE}(\%)=\frac{1}{N}\sum_{i=1}^{N}\left|\frac{\sigma_{\exp}-\sigma_{Model}}{\sigma_{\exp}}\right|\times 100\%$$

(24) $$R^{2}=\frac{\sum_{i=1}^{N}\left(\sigma_{\exp}-{\bar{\sigma }}_{\exp})(\sigma_{Model}-{\bar{\sigma }}_{Model}\right)}{\sqrt{\sum_{i=1}^{N}{\left(\sigma_{\exp}-{\bar{\sigma }}_{\exp}\right)}^{2}\sum_{i=1}^{N}{\left(\sigma_{Model}-{\bar{\sigma }}_{Model}\right)}^{2}}}$$

where $\sigma_{\exp}$, ${\bar{\sigma }}_{\exp}$, $\sigma_{Model}$, and ${\bar{\sigma }}_{Model}$ represent the experimental stress, the average experimental stress, the theoretical stress, and the average theoretical stress, respectively, and N is the number of data points.

**Figure 9:**
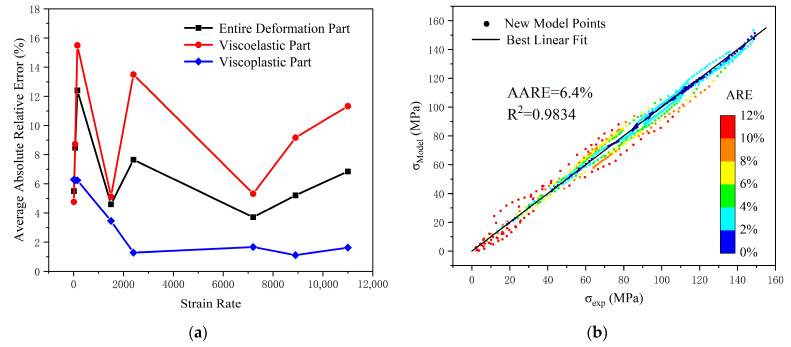
The AARE at different deformation stages and evaluation coefficients of the constitutive model’s predictive results. (**a**) AARE; (**b**) evaluation coefficients.

Figure 9 illustrates the relationship between the strain rate and the AARE at different deformation stages, as well as the evaluation coefficients of the constitutive model’s predictive results. Throughout the entire deformation stage, except for the AARE of 12.4% at a strain rate of 150 s^−1^, the errors under other strain-rate conditions are all less than 10%. In addition, the overall model’s R^2^ is 0.9834, and the AARE is 6.4%, both of which fully demonstrate the high accuracy of the constitutive model proposed. In the viscoelastic part, the AARE exhibits irregular fluctuations with rising strain rates. In the viscoplastic part, the AARE shows a trend of first decreasing and then stabilizing as the strain-rate increases, and the overall error value is relatively smaller compared to the viscoelastic part.

**Figure 10:**
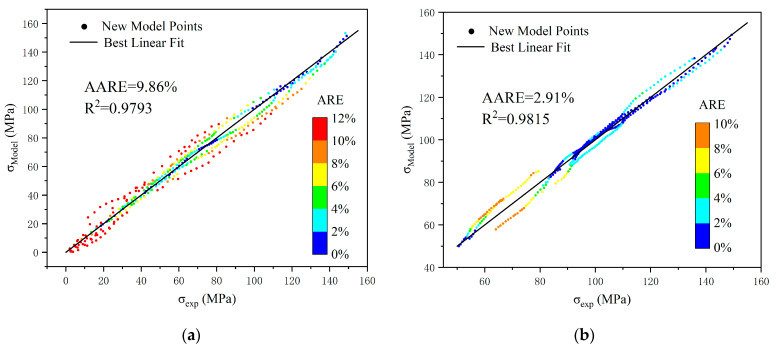
Evaluation coefficients of the constitutive model’s predicted results at various deformation parts. (**a**) Viscoelastic part; (**b**) viscoplastic part.

Figure 10 presents the evaluation coefficients of the constitutive model’s predicted results at various deformation parts. In the viscoelastic part, the R^2^ of the predicted results is 0.9793, and the AARE is 9.86%. The ARE between the theoretical and experimental results is relatively large in the initial and middle segments, while the error in the final segment is relatively small, which is related to the data weighting set during the fitting process. In the viscoplastic part, the R^2^ of the predicted results is 0.9815, and the AARE is 2.91%, with overall evaluation results superior to those in the viscoelastic part. Conversely, in the viscoplastic part, the error between the theoretical and experimental results is small in the initial and middle segments, while it is relatively large in the final segment. Although there are some variations in the accuracy of the constitutive model across various deformation stages, these differences are relatively small, indicating that the model has good stability throughout the entire deformation process and over a wide strain-rate range.

### 4.2. Application to Numerical Simulation

**Figure 11:**
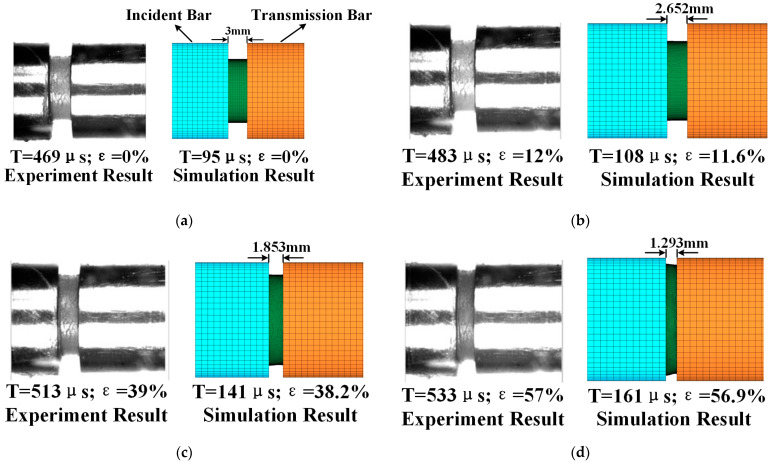

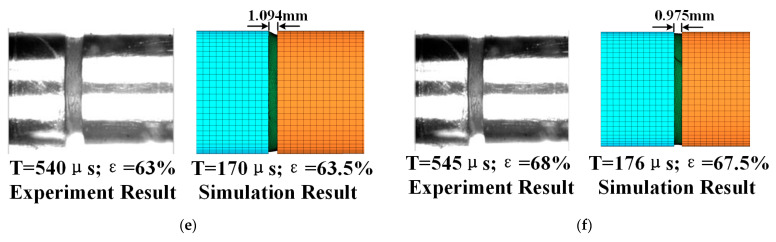
Comparison between the simulated compression process of the sample and the experimental results. (**a**) 0%; (**b**) 12%; (**c**) 39%; (**d**) 57%; (**e**) 63%; and (**f**) 68%.

Figure 11 presents a comparison between the simulated compression process of the sample and the experimental results. In the group of images, the left side represents the time recorded during the experimental process, while the right side corresponds to the simulation time. The time difference between the two is due to the difference in the striker bar’s impact distance. At 95 μs during the simulation process, the striker bar’s impact on the incident bar created an incident wave. This wave traveled to the specimen’s surface, causing it to compress. During the period from 95 μs to 176 μs, the specimen was progressively compressed in the direction of impact and gradually expanded radially. The simulated strain variation over time closely matched the experimental results.

**Figure 12:**
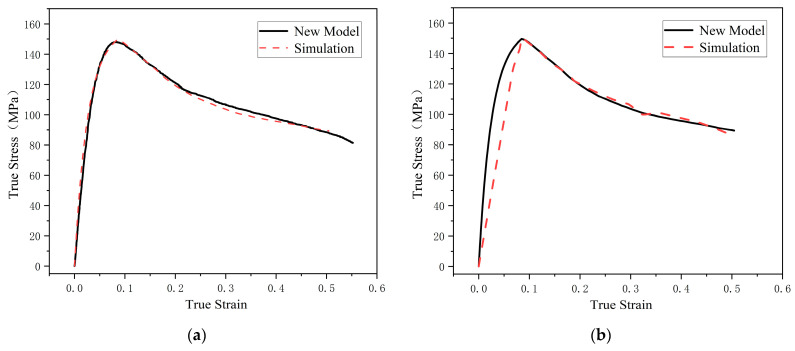
Comparison between the simulation results of the two material models and the theoretical results at a strain rate of 11,000 s^−1^. (**a**) MAT_89; (**b**) MAT_224.

Figure 12 presents a comparison between the simulation results of the two material models and the theoretical results at a strain rate of 11,000 s^−1^. It can be observed that material model MAT_224 achieves relatively high accuracy in the viscoplastic stage, but due to its linear calculation approach in the elastic stage, it cannot accurately describe the material’s viscoelastic properties. In contrast, material model MAT_89 demonstrates a high level of accuracy throughout the entire deformation process. These results verify that the suggested model can be used directly in numerical simulation software. Moreover, by defining different material models, it can be adapted to various simulation scenarios, thereby significantly reducing simulation time and effectively enhancing software stability.

## 5. Conclusions

Focusing on the mechanical properties of semi-crystalline polymers across a broad strain-rate spectrum, this study proposes a phenomenological constitutive model and conducts a systematic analysis of its structure, parameters, and application in simulation software. The following conclusions are drawn:

1. Through an analysis of the dynamic mechanical response characteristics of semi-crystalline polymers, a three-branch parallel constitutive model is put forward. This model precisely describes the complex mechanical responses caused by the combined impacts of strain hardening, strain-rate strengthening, and temperature softening.
2. By employing a hybrid global optimization algorithm in conjunction with experimental data from polypropylene, the optimal parameters for the proposed constitutive model were determined. Multidimensional evaluations indicate that the model possesses high accuracy, with a coefficient of determination reaching 0.9834 and an average absolute relative error controlled within 6.4%.
3. A polypropylene SHPB numerical simulation model was developed. The software database’s two material models precisely reflect the proposed constitutive model’s mechanism. The simulation and theoretical model’s high consistency shows that the constitutive model and simulation software are highly compatible and that the model is highly applicable. Consequently, it can provide high-confidence material models for various high-strain-rate simulation scenarios.

## Figures and Tables

**Table 1:** The coefficients of the Ree–Eyring model.

$\sigma_{0}$ (MPa)	$\frac{2kT}{V}$ (MPa)	γ	${\dot{\varepsilon }}_{0}$ (s^−1^)
69.4135	0.156291	0.370445	1

**Table 2:** The coefficients of determination for each deformation stage under different weight settings.

Fitting Weight	Overall Deformation	Viscoelastic Segment	Viscoplastic Segment
0	0.9861	0.9873	0.9690
20	0.9834	0.9793	0.9815
100	0.9813	0.9761	0.9853

**Table 3:** The coefficients of the constitutive model in the viscoelastic part.

$E_{\infty }^{ve}$ (MPa)	$\eta_{1_{0}}$ (MPa·s)	$\eta_{2_{0}}$ (MPa·s)	$\alpha_{1}$	$\alpha_{2}$	$\theta_{1_{0}}$ (s)	$\theta_{2_{0}}$ (s)	$\beta_{1}$	$\beta_{2}$
72.2408	1.05483	56.7945	0.584895	0.940577	0.110474	0.0277132	1.18157	0.999395

**Table 4:** The coefficients of the constitutive model in the viscoplastic part.

a	*b*_1_		*b*_2_		*c*_1_		*c*_2_		*d*_1_		*d*_2_
−199.958	−0.849211		1999.91		0.790387		2.6873		2427.98		276.027
***e*_1_**		***e*_2_**		***T*_0_ (K)**		***β_p_***		***ρ* (g/cm^3^)**		***C_v_*** **(J/kg·K)**	
1.2935		1.80688		298		0.9		0.874		1900	

## Data Availability

The original contributions presented in this study are included in the article. Further inquiries can be directed to the corresponding authors.

## Nomenclature

$\sigma^{ve}$	total deviatoric stress in viscoelastic stage
$\sigma_{e}^{ve}$	deviatoric stress of the non-Maxwell branch
$\sigma_{M_{i}}^{ve}$	deviatoric stress of the Maxwell branch
$\sigma_{y}$	yield stress
$\sigma_{0}$	reference yield stress
$\varepsilon^{ve}$	total elastic strain
$\varepsilon_{e}^{ve}$	elastic strain of the non-Maxwell branch
$\varepsilon_{M_{i}}^{ve}$	elastic strain of the Maxwell branch
$\varepsilon_{v_{i}}^{ve}$	elastic strain of the viscous element
$E_{\infty }^{ve},\;E_{\infty }^{vp}$	elastic moduli in non-Maxwell branch
$E_{i}^{ve}$	elastic moduli in Maxwell branch
$E_{\infty_{0}}$	reference elastic modulus
$\dot{\varepsilon }$	strain rate
${\dot{\varepsilon }}_{v_{i}}^{ve}$	strain-rate of the viscous elements
${\dot{\varepsilon }}_{0}$	reference strain rate
$\eta_{i}^{ve},\eta_{i}^{vp}$	viscosity
$\eta_{i_{0}}$	reference viscosity
t	deformation time
$\theta_{i}^{ve},\;\theta_{i}^{vp}$	relaxation time
$\theta_{i_{0}}$	reference relaxation time
$\alpha_{i}$	strain-rate coefficients
$\beta_{i}$	strain-rate coefficients
k	Boltzmann constant
T	material temperature
$T_{0}$	initial temperature
$T_{m}$	melting temperature
V	activation volume
γ	yield stress coefficients
a	temperature coefficients
$b_{i}$	temperature coefficients
$c_{i}$	temperature coefficients
$d_{i}$	temperature coefficients
$e_{i}$	temperature coefficients
$\beta_{p}$	Taylor–Quinney factor
ρ	density
Cv	specific heat capacity at constant volume
$\sigma_{\exp}$	experimental stress
${\bar{\sigma }}_{\exp}$	average experimental stress
$\sigma_{Model}$	theoretical stress
${\bar{\sigma }}_{Model}$	average theoretical stress
N	number of data points
